# Investigation on Carbonation and Permeability of Concrete with Rice Hush Ash and Shop Solution Addition

**DOI:** 10.3390/ma15176149

**Published:** 2022-09-05

**Authors:** Manish Kumar, Ashutosh Anand, Rajeshwari Chatterjee, Shubham Sharma, Tushar Kanti Maiti, Shashi Prakash Dwivedi, Ambuj Saxena, Changhe Li, Elsayed Mohamed Tag Eldin

**Affiliations:** 1 Department of Civil Engineering, GD Goenka University, Gurugram 122103, India; 2 Department of Electronics and Communication Engineering, Presidency University, Bangalore 560064, India; 3 Department of Hotel Management & Catering Technology, Birla Institute of Technology Mesra, Ranchi 835215, India; 4 Department of Mechanical Engineering, IK Gujral Punjab Technical University, Main Campus-Kapurthala, Kapurthala 144603, India; 5 Mechanical Engineering Department, University Center for Research & Development, Chandigarh University, Mohali 140413, India; 6 Department of Polymer and Process Engineering, IIT Roorkee, Saharanpur Campus, Saharanpur 247001, India; 7 G.L. Bajaj Institute of Technology & Management, Greater Noida 201310, India; 8 School of Mechanical and Automotive Engineering, Qingdao University of Technology, Qingdao 266520, China; 9 Faculty of Engineering and Technology, Future University in Egypt, New Cairo 11835, Egypt

**Keywords:** rice husk ash, soap solution, carbonation, permeability, SEM, EDX

## Abstract

The goal of this study was to determine the coefficient of permeability as well as the rate of carbonation of concrete constructed with rice husk ash (RHA) as a partial replacement for cement (i.e., 5%, 10%, and 15%) and two different concentrations of soap solutions (i.e., 1 percent and 2 percent). The microstructural studies of RHA, and carbonated samples have been conducted by using Scanning Electron Microscope (SEM) and X-Ray Diffraction (XRD) analysis. According to this study, the carbonation depth of concrete made with 1% and 2% soap solution concentration and without rice husk ash decreased by 11.89% and 46.55%, respectively. From the results, it may also be observed that the carbonation depth of concrete made with up to 10% replacement of cement by rice husk ash led to maximum carbonation resistance, while more than 10% replacement of cement showed higher carbonation depth. It is also observed that the coefficient of permeability of concrete with 2% soap solution significantly decreased as compared to the 1% soap solution and control mix. It may be observed from the SEM images that 0% soap solution (M1) concrete has a very rough concrete surface which may indicate more voids. However, 2% soap solution concrete has a much smoother surface, which indicates a smaller number of voids. Furthermore, the SEM images showed that the soap solution helps in filling the voids of concrete which ultimately helps in reduction in permeability. Energy Dispersive X-Ray Analysis (EDX) of concrete with 0% (M1) and 2% (M6) soap solution disclosed that the concrete with 2% soap solution (M6) exhibited more silica element formation than the concrete with no soap solution (M1).

## 1. Introduction

Durability is one of the important parameters that must be taken into consideration during the construction of reinforced concrete (RC) structures. The permeability and carbonation of concrete are the two major parameters that lead to the corrosion of reinforcement in RC structures. These days, the world is more concerned about reducing the generation of CO_2_ gas as the production of one ton of cement produces one ton of CO_2_ gas [1]. Utilization of mineral admixtures in making concrete is known to enhance the durability property of the RC structures [2]. In contrast, when CO_2_ gas from air reacts with Ca(OH)_2_ in concrete and form calcium carbonate, this process is called carbonation [3]. Because of carbonation the alkalinity of the pore water decreases which increases the rate of corrosion in steel reinforcement. Carbonation is also an important factor in terms of the durability of concrete. Carbonation has no impact on the mechanical properties of concrete but it reduces the pH of concrete which leads to initiation of corrosion of steel reinforcement [2]. In carbonation, CO_2_ converts free lime into CaCO_3_ and water thereby reducing the pH. Carbonation occurs in concrete because the calcium bearing phases present are attacked by carbon dioxide of the air and converted to calcium carbonate. When the pH of concrete is reduced to about 9 or below, the passive layer of the steel reinforcement is destroyed and corrosion of the steel takes place [3].

Rice husk ash is an industrial waste of the rice industry and dumping of RHA is also a major problem for the industry. The partial replacement of cement by RHA reduces the dependency on cement as RHA is a rich source of silica. It also helps in reducing the problem of RHA dumping [4]. When paddy is processed in rice mills, about 78% of rice is the final produce while the remaining 22% is perceived as husk. The burning of husk produces about 25% of husk ash by weight [5]. The particle size of RHA also has a major impact on the mechanical properties of concrete [3,4,5]. Habeeb and Fayyadh [6] had investigated the influence of RHA average particle size on properties of concrete and they observed that at early ages the strength was comparable, while at the age of 28 days, the finer RHA with size less than 90 micron exhibited higher strength than the sample with coarser RHA. Cement paste contains 25–50 wt% calcium hydroxide (Ca(OH)_2_), which results in the pH 12.5 for fresh concrete [7,8]. Concrete without voids is very difficult to prepare. Mario C. Lombardo et al. [9] had prepared a waterproof concrete and mortar, and suggested that waterproofing can be achieved by use of micro particles in concrete mixtures which may fill the voids inside the concrete.

Ozturk et al. [10] revealed that the use of RHA as well as other pozzolanic compounds may reduce CO_2_ emissions by 25% while increasing cost efficiency by 65% throughout the concrete manufacturing process. The study’s results show that building practices have become more environmentally friendly, and also point to potential new avenues for RHA’s waste management.

Vishavkarma and Harish [11] have checked the influence of RHA on the chloride permeability, water absorption, and permeable pore space of cementitious mortar. They observed that as RHA content increased in the mortar, permeability, water absorption, and permeable pore spaces reduced whereas compressive strength enhanced. Water absorption and permeable pore space were found to have a strong correlation, as well as permeable pore space and the amount of charge that was allowed to flow through.

According to Abdila et al. [12] the use of fly ash and ground granulated blast furnace slag (GGBFS) geopolymers can support soil stabilization through improving strength. A previous study solely employd fly ash or GGBFS; however, the strength value did not exceed the ASTM D 4609 (<0.8 MPa) criterion for soil-stabilization in road construction applications. A UCS test on stabilized soil samples was described. The article concludes that GGBFS and fly-ash-based geo-polymers may be utilized to stabilize soil.

Amin MN et al. [13] stated that reducing cement demand and preserving natural resources will be achieved by using RHA as a replacement for fine aggregate and cement in the construction industry, which will result in green building. More research is needed before RHA’s efficacy may be expanded for the bulk of its uses. Mareike et al. [14] observed that concrete made with RHA performed better against durability parameters namely, carbonation resistance and capillary suction mainly due to the dense matrix formed by RHA and cement.

Edward Scripture et al. [15] had prepared a composition for waterproof concrete and mortar. According to them, concrete can made waterproof by two different methods. The first method involves only small amount of water, which is required for hydration purpose, which is practically not very difficult to achieve. In the second method, the voids have been filled by the extra water in the concrete. They used a water repellent agent with cement during the preparation of the concrete mix, and assumed that these water repellent agents deposited either in voids or near the voids. In a later stage, the voids repel the water which comes into the concrete through capillary action because water repellent agents are present which do not allow the water to enter. They used steric acid as water repellent agent. They also suggested that soap solution or different fatty acids could also be used as they have similar properties to that of steric acid. Khanna [16] has also suggested that soap solution may be used as waterproofing. Therefore, in the present work a study has been carried out to study carbonation and permeability of concrete made with different concentrations of soap solution and rice husk content. Locally available fatty acid soap was used for preparation of soap solution having the property to attach itself with dust and make a hydrophobic condition around the particle. Concrete prepared with soap solution as hydrophobic agent and finely divided filler material (rice husk ash) as stuffing can make the concrete waterproof.

In this paper, the problem of carbonation has been tackled by replacing the cement with RHA at different concentrations of the soap solution. The effect of replacement of cement by RHA at different soap solution concentrations on the compressive strength of concrete structure was studied. The effect on carbonation and permeability of concrete structure also been discussed in this paper at different concentrations of soap solution and at different ratios of RHA.

## 2. Materials and Methods

### 2.1. Material Used

**Figure 1:**
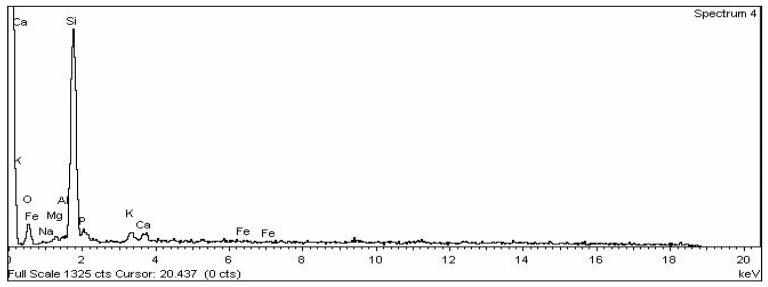
EDX analysis graph of RHA.

**Figure 2:**
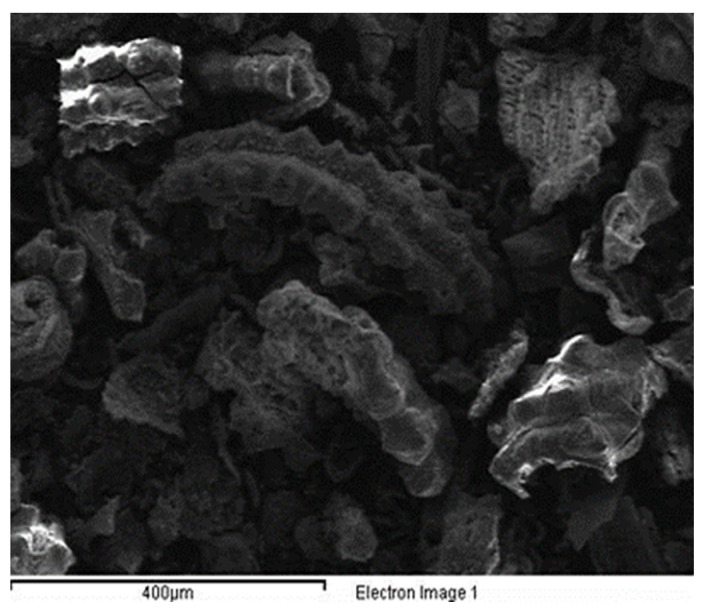
SEM Image of RHA.

The ordinary Portland cement (43 grade) cement was used, satisfying Indian standards IS: 8112-1989 [17]. Soaps are water soluble sodium or potassium salts of fatty acids. Fatty acids are merely carboxylic acid with long hydro carbon chains [6]. Soap used in this project is produced locally and its classification is given in Table 1. This soap was used with normal water in different concentrations in this research work. Rice husk ash was taken from the local rice industry in dry condition. EDX analysis was done on rice husk ash as shown in Figure 1 and the results of EDX analysis are presented in Table 2. The rice husk ash of particle size passing through a 300-micron sieve was used. The SEM image of rice husk ash is shown in Figure 2. The zone II fine aggregates and coarse aggregate with mean size of aggregate (MSA) 20 mm was used in the present work which is as per Indian standard IS 383 [13]. The nature of the used aggregates is quartz. The sieve analysis had been performed to separate the zone II fine aggregates. The specific gravity of fine and coarse aggregate was 2.61 and 2.67 respectively, which was measured by pycnometer in the laboratory.

### 2.2. Preparation of Sample and Test Procedure

Mix design as per IS 10262:2009 [18] of M20 grade concrete with W/C ratio 0.5, cement content 320 kg/m^3^, fine aggregate 732.94 kg/m^3^ and coarse aggregate 1223.34 kg/m^3^ was used. Concrete cubes of size 150 × 150 × 150 mm were prepared with three replicates for two concentrations of soap solution (i.e., 1% and 2% by weight) and different replacement levels of rice husk ash in place of cement (i.e., 0%, 5%, 10% and 15% by weight). Soap solution of concentration 1% and 2% was prepared by weight with pH value 12.

The slump value of the concrete was kept between 50 mm to 65 mm using suitable doses of MasterGlenium ACE 30 super plasticizer [19], which is 0.85% of binder weight

### 2.3. Methods

#### 2.3.1. Carbonation Test

Concrete cubes were taken out from the curing tank at the age of 7 and 28 days and compressive strength of the cube was tested for each set as per IS 516:1959 [20]. After 28 days moist curing, the remaining cubes were kept in laboratory condition for 14 days before carbonation and permeability testing. After 14-day laboratory conditioning, the concrete cube was kept in an accelerated carbonation environment as per EN code [21]. Accelerated carbonation environment was maintained with carbon dioxide concentration = 4% ± 0.5%, RH = 55 ± 5% and temperature = 20 °C. The CO_2_ cylinder was attached to the carbonation chamber. After 70 days of accelerated carbonation exposure, carbonation depth on each concrete cube was measured by splitting the cube into two halves and spraying the 1N phenolphthalein indicator on the new split surface. This indicator changed the color of the uncarbonated part to dark pink whereas the carbonated part was colorless. The carbonation rate coefficient was calculated from the results of carbonation depth using the square root equation as given in Equation (1).

(1) $$x=k\sqrt{t}$$

where ‘*x*’ is depth of carbonation, ‘*t*’ is time of accelerated carbonation exposure and ‘*k*’ is carbonation rate coefficient.

#### 2.3.2. Permeability Test

The permeability of the concrete cubes was also measured at the age of 42 days (28 days moist curing followed by 14 days laboratory conditioning) [22]. As per the guidelines given in IS code 3085 (1965), the flow of water through the concrete in saturated conditions was achieved thereafter, measuring the amount water collected for a particular interval of time. Coefficient of permeability was then calculated by using formula as:

(2) $$k=\frac{Q}{A\times T\times \frac{H}{L}}\;\;\;\;$$

where *k* is coefficient of permeability, *Q* is discharge in ml, *A* is area of specimen, *T* is time in sec, *H* is head of water and *L* is length of specimen.

## 3. Result and Discussions

**Figure 3:**
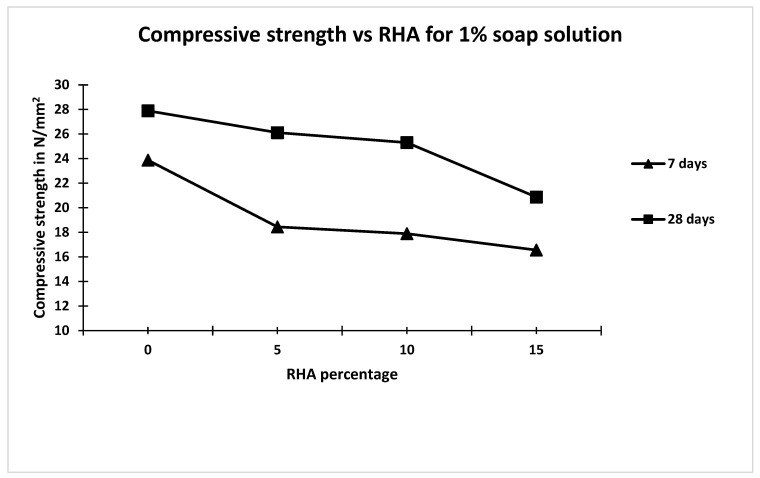
Graph between compressive strength and RHA for 1 % soap solution.

**Figure 4:**
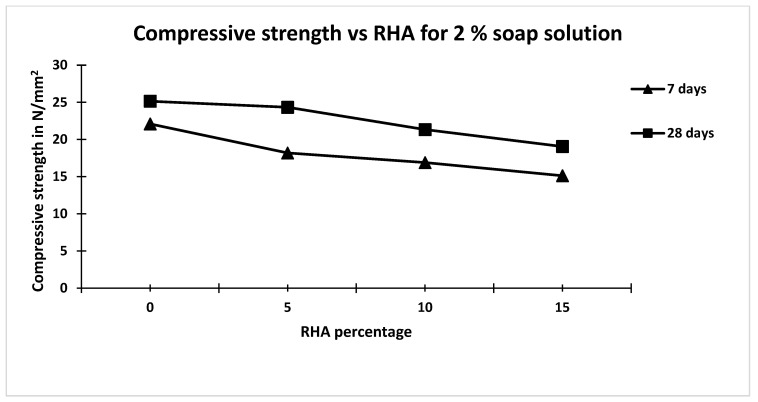
Graph between compressive strength and RHA for 2 % soap solution.

Compressive strengths of different mixes at 7th and 28th day for 1% and 2% soap solution and different percentages of rice husk ash are given in Table 3. From Table 3, it is observed that with an increase in replacement of cement by RHA (passed through a 300-micron sieve). the compressive strength decreased irrespective of the concentration of soap solution. From Table 3, it is also observed that the compressive strength of concrete made with 1 percent soap solution is higher than the concrete made with 2 percent soap, for all percentage replacements of cement with RHA. Further, it is observed from Table 3 that the 28 days compressive strength of concrete made with 1% soap solution and without rice husk ash was 8.4% less as compared to concrete made without soap solution and without rice husk [23,24,25,26]. Similarly, from Table 4, it is also observed that the 28 days compressive strength of concrete made with 2 % soap solution and without rice husk ash is 15% less compared to that made without soap solution and without rice husk. The percentage decreases in 28 days compressive strength of concrete made with different percentage of rice husk ash, i.e., 5%, 10% and 15% as compared to that made with 1% soap solution and without rice husk ash, are 6.38%, 9.28% and 25.87% respectively [27,28,29]. Similarly, the percentage decrease in 28 days compressive strength of concrete made with different percentages of rice husk ash, i.e., 5%, 10% and 15%, compared to that made with 2% soap solution and without rice husk ash are 3.22%, 15.15% and 24.22%, respectively [30,31,32,33,34]. Hence, it is clear that replacement of cement up to 10% by rice husk ash has no significant effect on compressive strength of concrete irrespective of soap solution concentration for 7 and 28 days. Results of 7 days and 28 days compressive strength of concrete are plotted against percentage of rice husk ash for 1% and 2% soap solution and are shown in Figure 3 and Figure 4.

**Figure 5:**
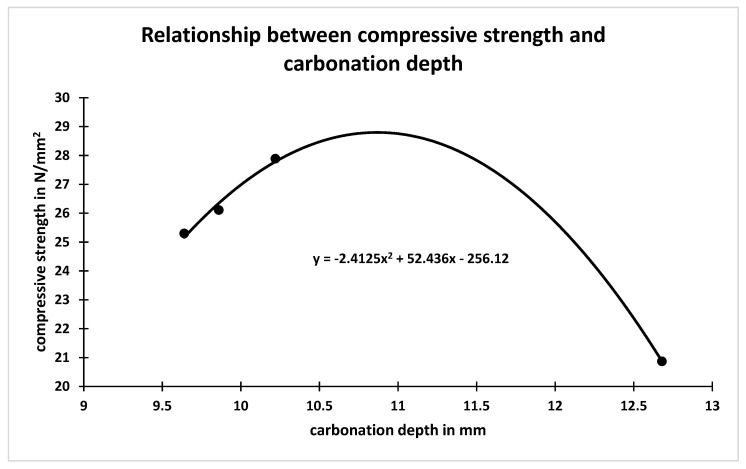
Relationship between compressive strength and carbonation depth.

**Figure 6:**
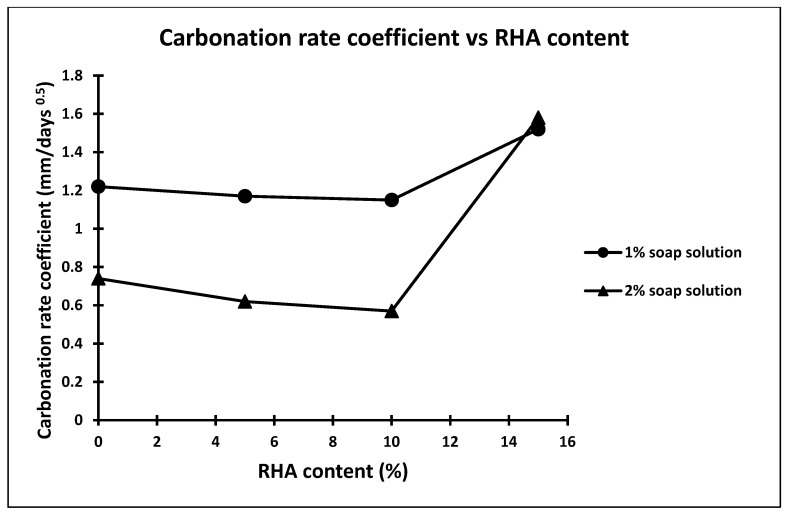
Carbonation rate coefficient *v*/*s* RHA.

The carbonation depth of the concrete made with all mix combinations were determined at the accelerated carbonation exposure age of 70 days through spraying phenolphthalein solution on the split surface as depicted in Figure 5. The result of carbonation depth and coefficient of permeability of all concrete mixes are presented in Table 4. The coefficient of permeability of concrete for all mix combinations was calculated from the amount of water collected for a particular interval of time after attaining saturated flow at pressure 8 kg/cm^2^. The carbonation rate coefficient was calculated using square root formulae. From Table 4, it is observed that carbonation depth of the concrete made with soap solution concentration (i.e., 1% and 2%) decreases to that of concrete made without soap solution. The carbonation depth of concrete made with 1% and 2% soap solution concentration decreased 1 by 1.89% and 46.55%, respectively, compared to that of concrete made without soap solution [35,36,37,38,39,40,41,42]. Further, from Table 4, it is also observed that the change in the carbonation depth of concrete made with partial replacement of cement by different percentages of RHA, i.e., 5%, 10% and 15%, compared to that of concrete made with 1% and 2% soap solution concentration and without RHA are 4.09% and 5.73%, −24.59% and 16.2%, and 22.9% and −113.51%, respectively. A plot made between carbonation rate coefficient of concrete versus different percentage RHA for 1 and 2 percent of soap solution is shown in Figure 6. From Figure 6, it is observed that the carbonation rate coefficient of concrete decreases with increase in replacement percentage of cement by RHA up to 10%, irrespective of soap solution concentration [35,36,37,38,39,40,41,42,43,44,45,46,47,48,49]. Further, more than 10% replacement of cement by RHA results increase in the carbonation rate coefficient of concrete irrespective of soap solution concentration, as shown in Figure 6.

**Figure 7:**
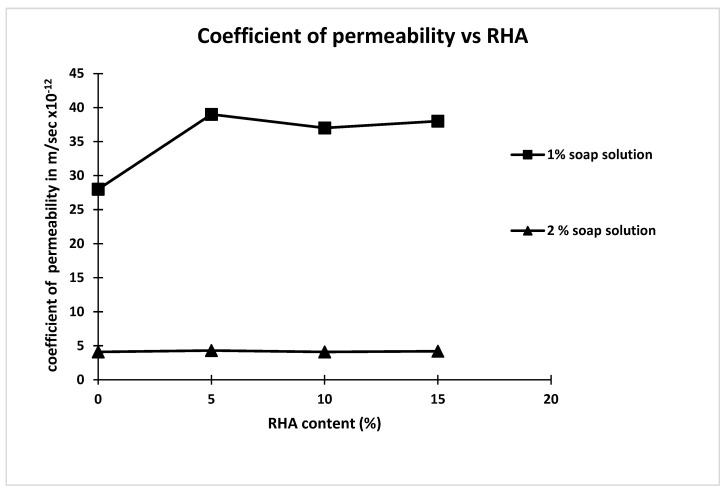
Coefficient of permeability vs. RHA for 1 and 2 percent soap solution.

The coefficients of permeability of concrete mixes M2 and M6 are 3.44% and 85.86% compared to that of concrete M1, respectively. From Table 4, it is also observed that the coefficient of permeability of concrete for 1% soap solution increases with increasing percentage of rice hush ash content. It is also observed from Table 4 that the coefficient of permeability of concrete for 2% soap solution significantly decreases with increasing percentage of rice hush ash content. Further, from Table 4, it is also observed that the change in the coefficient of permeability of concrete made with partial replacement of cement by different percentages of RHA of 5%, 10% and 15%, compared to that concrete made with 1% and 2% soap solution concentration and without RHA, are −34.48%, −27.58% and −31.03%, and 85.17%, 85.86% and 85.51%, respectively [50,51,52,53,54]. It is observed that the coefficient of permeability of concrete with 1% soap solution showed negligible change whereas the coefficient of permeability of concrete with 2% soap solution was reduced by 85.86% compared to mix M1. The coefficient of permeability of concrete made with 2% soap solution showed a significant reduction compared to mix M1 and this may be due to the hydrophobic action of soap solution in concrete which repels the water ingress. The coefficient of permeability of concrete made with different percentages of RHA (5%, 10% and 15%) compared to that of concrete made with 1% soap solution shows higher coefficient of permeability whereas when compared with 2 percent soap solution no significant change is observed [52,53,54,55,56,57,58,59]. A plot is made between coefficient of permeability of concrete and different percentage RHA for 1 and 2 percent of soap solution as shown in Figure 7. From Figure 7, it is observed that the coefficient of permeability of concrete for 2 percent of soap solution showed negligible change with increasing percentage of RHA content. However, the coefficient of permeability of concrete for 1 percent of soap solution increased for concrete made with 5% RHA content (M3) compared to that made without RHA content (M2) and beyond 5% RHA content no significant change in coefficient of permeability was observed.

The above-mentioned observation may due to the effect of soap solution in concrete mixture. Soap is a water reducing agent and it makes the cement surface particles hydrophilic after absorption. HO-C-H, O-H, COOH, HO-C-C=O are the different active groups in the molecule which promote this adsorption. These agents are mainly anionic, which gives a negative potential to the particles. This results in the orientation of the water dipole, thus facilitating its mobility due to the prevention of close approach to particles. Soap also influences the form of crystallization products of hydration, the rate of hydration and the establishment of a rigid structure in the cement paste. The properties of the absorbed layer at the surface particle reduces the water content. The water reducing agent does not change capillary structure but decreases the water binder ratio and gives the desired flow.

**Figure 8:**
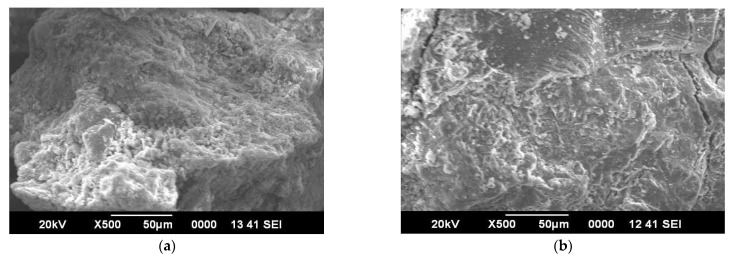
(**a**) SEM image of M1; (**b**) SEM image of M6.

**Figure 9:**
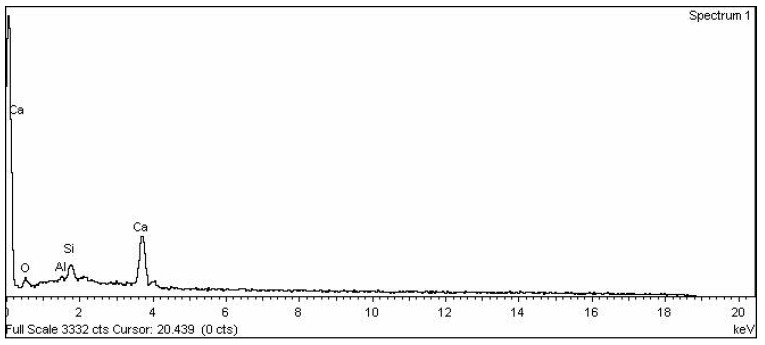
EDX analysis of concrete powder with 0% soap solution and RHA.

**Figure 10:**
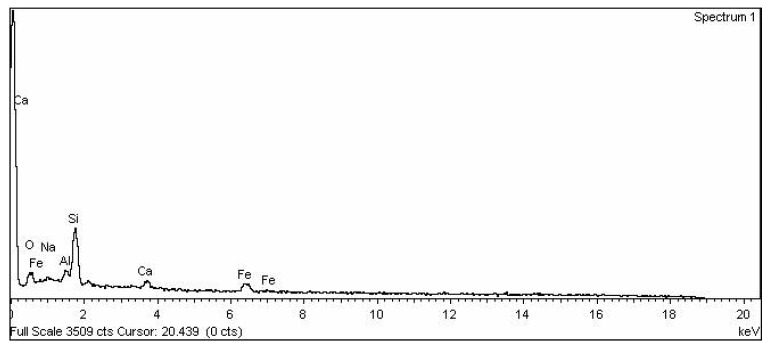
EDX analysis of concrete powder with 2% soap solution and RHA.

SEM images of M1 and M6 are shown in Figure 8a,b, respectively. When the SEM image of zero percent soap solution(M1) and 2 percent soap solution(M6) concrete are observed, it is found that concrete without soap solution has a very rough surface which may indicate more voids. In contrast, the surface of concrete with 2 percent soap solution is very much smoother and has much less visible voids. From the permeability result, it is also found that coefficient of permeability with 2 percent soap solution is very much less in comparison with 0 percent soap solution. From the observation of SEM images, it can be said that soap solution helps in filling the voids of concrete which ultimately helps in reduction in permeability [52,53,54,55,56,57,58,59,60,61,62,63,64,65,66,67,68,69,70]. EDX analyses of concrete with 0% (M1) and 2 % (M6) soap solution are shown in Figure 9 and Figure 10 respectively. From the EDX analysis also, it is found that the concrete with 2% soap solution (M6) showed more silica element formation than the concrete with no soap solution (M1) [70,71,72,73].

## 4. Conclusions

From the result of compressive strength, it is observed up to 10 percent replacement of cement by rice husk ash showed negligible variation in 28 days compressive strength compared to that of the control mix irrespective of soap solution concentration. Moreover, it was found that:

i The 28 days compressive strength of concrete containing 1% and 2% soap solution decreased by 8.4% and 15% compared to that made without soap solution and RHA.
ii The carbonation depth of concrete made with 1% and 2% soap solution concentration and without rice husk ash decreased 11.89% and 46.55%, respectively.
iii The carbonation depth of concrete made with up to 10% replacement showed maximum carbonation resistance, while more than 10% replacement of cement showed higher carbonation depth.
iv The coefficient of permeability of concrete for 2% soap solution significantly decreased compared to that 1% soap solution and control mix.
v Increasing the percentage of rice hush ash content has a negligible effect on the coefficient of permeability of concrete made with 2% soap solution concentration.
vi From these results, it is concluded that the reduction in coefficient of permeability is mainly because of the soap solution.
vii The rice husk ash carbonation depth has been reduced but only up to 10%, and any further replacement increases the carbonation depth.

## Figures and Tables

**Table 1:** Chemical classification of soap.

PH	Moisture	Free Fatty Acid as Oleic Acid (C_18_H_34_O_2_)	Chlorides	Alcohol Insoluble	Total Alkalinity as NaOH
11.5	9.3%	37.2%	0.3%	37%	7.2%

**Table 2:** Elemental analysis of RHA.

Element	Weight%	Atomic%
O	34.07	48.47
Na	0.13	0.13
Mg	1.39	1.30
Al	1.21	1.02
Si	51.61	41.82
P	4.27	3.14
K	3.70	2.15
Ca	3.09	1.76
Fe	0.53	0.22

**Table 3:** Different composition of concrete mixes.

Concrete Mix	Soap Solution Concentration	RHA Percentage
M1	0	0
M2	1	0
M3	1	5
M4	1	10
M5	1	15
M6	2	0
M7	2	5
M8	2	10
M9	2	15

**Table 4:** Carbonation depth, carbonation rate coefficient and coefficient of permeability of different concrete mixes.

Concrete Mix	Soap Solution Concentration	RHA Percentage	Carbonation Depth mm	Carbonation Rate Coefficient (K) mm/days^0.5^	Coefficient of Permeability m/s
M1	0	0	11.6	1.38	2.9 × 10^−11^
M2	1	0	10.22	1.22	2.8 × 10^−11^
M3	1	5	09.86	1.17	3.9 × 10^−11^
M4	1	10	09.64	1.15	3.7 × 10^−11^
M5	1	15	12.68	1.52	3.8 × 10^−11^
M6	2	0	6.2	0.74	4.1 × 10^−12^
M7	2	5	5.18	0.62	4.3 × 10^−12^
M8	2	10	4.78	0.57	4.1 × 10^−12^
M9	2	15	13.25	1.58	4.2 × 10^−12^

## Data Availability

No data were used to support this study.

## References

[1] Kumar M., Malay N., Kujur J. (2018). Study of natural carbonation of concrete incorporating marble dust. Proc. Inst. Civ. Eng.-Constr. Mater..

[2] Kumar M., Sinha A.K., Kujur J. (2021). Mechanical and durability studies on high volume fly-ash concrete. Struct. Concr..

[3] Kumar M., Kujur J., Chatterjee R., Chattopadhyaya S., Sharma S., Dwivedi S.P., Saxena A., Rajkumar S., Anand A. (2022). Corrosion Zones of Rebar in High-Volume Fly-Ash Concrete through Potentiodynamic Study in Concrete Powder Solution Extracts: A Sustainable Construction Approach. Adv. Civ. Eng..

[4] Dongmin A., Yupeng G., Yanchao Z., Zichen W. (2010). A green route to preparation of silica powders with rice husk ash and waste gas. Chem. Eng. J..

[5] Devi T.K., Chanu N.M. (2013). Contribution of rice husk ash to the properties of cement mortar and concrete. Int. J. Eng. Res. Technol..

[6] Habeeb G.A., Fayyadh M.M. (2009). Rice Husk Ash Concrete: The Effect of RHA Average Particle Size on Mechanical Properties and Drying Shrinkage. Mater. Sci. Eng..

[7] Arredondo-Rea S.P., Corral-Higuera R., Gómez-Soberón J.M., Castorena-González J.H., Orozco-Carmona V., Almar-al-Sánchez J.L. (2012). Carbonation rate and reinforcing steel corrosion of concretes with recycled concrete aggregates and supplemen-tary cementing materials. Int. J. Electrochem. Sci..

[8] Sulapha P., Wong S.F., Wee T.H., Swaddiwudhipong S. (2003). Carbonation of Concrete Containing Mineral Admixtures. J. Mater. Civ. Eng..

[9] Lombardo M.C. (1966). Densifier and Waterproofing Agents for Mortar and Concrete and Method of Making same.

[10] Ozturk E., Ince C., Derogar S., Ball R. (2022). Factors affecting the CO_2_ emissions, cost efficiency and eco-strength efficiency of con-crete containing rice husk ash: A database study. Constr. Build. Mater..

[11] Vishavkarma A., Harish K.V. (2022). Effect of rice husk ash on permeation characteristic of cementitious mortar. Mater. Today Proc..

[12] Abdila S.R., Abdullah M.M., Ahmad R., Nergis D.D.B., Rahim S.Z., Omar M.F., Sandu A.V., Vizureanu P. (2022). Potential of soil stabilization using ground granulated blast furnace slag (GGBFS) and fly ash via geopolymerization method: A Review. Materials.

[13] Amin M.N., Ahmad W., Khan K., Sayed M.M. (2022). Mapping research knowledge on rice husk ash application in concrete: A sci-entometric review. Materials.

[14] Thiedeitz M., Ostermaier B., Kränkel T. (2022). Rice husk ash as an additive in mortar–Contribution to microstructural, strength and durability performance. Resour. Conserv. Recycl..

[15] Scripture E.W. (1942). Waterproofing Composition for Concrete and Mortar.

[16] Khanna P.N. (2019). Indian Practical Civil Engineers.

[17] (2013). Specification for 43 Grade Ordinary Portland Cement.

[18] (1970). Specification for Coarse and Fine Aggregates.

[19] (2009). Concrete Mix Design.

[20] (1999). Specification for Concrete Admixtures.

[21] (1959). 1959 for Methods of Tests for Strength of Concrete.

[22] (2008). Draft v8 December 2008 Testing Hardened Concrete—Part XX: Determination of the Car-Bonation Resistance of Concrete: Accelerated Carbonation Method, prCEN/TS 12390-XXX:2008 (E). https://standards.iteh.ai/catalog/standards/sist/da305181-51c1-4f10-88e2-20424af56a72/sist-en-12390-12-2020.

[23] (1965). 1965 for Coefficient of Permeability of Concrete.

[24] Ilyas R.A., Zuhri M.Y.M., Norrrahim M.N.F., Misenan M.S.M., Jenol M.A., Samsudin S.A., Nurazzi N.M., Asyraf M.R.M., Supian A.B.M., Bangar S.P. (2022). Natural Fiber-Reinforced Polycaprolactone Green and Hybrid Biocomposites for Various Advanced Applications. Polymers.

[25] Ilyas R.A., Zuhri M.Y.M., Aisyah H.A., Asyraf M.R.M., Hassan S.A., Zainudin E.S., Sapuan S.M., Sharma S., Bangar S.P., Jumaidin R. (2022). Natural Fiber-Reinforced Polylactic Acid, Polylactic Acid Blends and Their Composites for Advanced Applications. Polymers.

[26] Sharma S., Patyal V., Sudhakara P., Singh J., Petru M., Ilyas R.A. (2021). Mechanical, morphological, and fracture-deformation behavior of MWCNTs-reinforced (Al–Cu–Mg–T351) alloy cast nanocomposites fabricated by optimized mechanical milling and powder metallurgy techniques. Nanotechnol. Rev..

[27] Chohan J.S., Mittal N., Kumar R., Singh S., Sharma S., Dwivedi S.P., Saxena A., Chattopadhyaya S., Ilyas R.A., Le C.H. (2021). Optimization of FFF process parameters by naked mole-rat algorithms with en-hanced exploration and exploitation capabilities. Polymers.

[28] Ilyas R.A., Sapuan S.M., Asyraf M.R.M., Dayana D.A.Z.N., Amelia J.J.N., Rani M.S.A., Norrrahim M.N.F., Nurazzi N.M., Aisyah H.A., Sharma S. (2021). Polymer composites filled with metal derivatives: A review of flame retardants. Polymers.

[29] Chohan J.S., Mittal N., Kumar R., Singh S., Sharma S., Singh J., Rao K.V., Mia M., Pimenov D.Y., Dwivedi S.P. (2020). Mechanical Strength Enhancement of 3D Printed Acrylonitrile Butadiene Styrene Polymer Components Using Neural Network Optimization Algorithm. Polymers.

[30] Singh Y., Singh J., Sharma S., Aggarwal V., Pruncu C.I. (2021). Multi-objective optimization of kerf-taper and sur-face-roughness quality characteristics for cutting-operation on coir and carbon fibre reinforced epoxy hybrid polymeric com-posites during CO_2_-pulsed laser-cutting using RSM. Lasers Manuf. Mater. Process..

[31] Sharma S., Singh J., Kumar H., Sharma A., Aggarwal V., Gill A.S., Jayarambabu N., Kailasa S., Rao K.V. (2020). Utilization of rapid prototyping technology for the fabrication of an orthopedic shoe inserts for foot pain reprieve using thermo-softening viscoelastic polymers: A novel experimental approach. Meas. Control.

[32] Singh Y., Singh J., Sharma S., Sharma A., Chohan J.S. (2022). Process parameter optimization in laser cutting of coir fiber re-inforced epoxy composite—A review. Mater. Today Proc..

[33] Chohan J.S., Kumar R., Singh T.H.B., Singh S., Sharma S., Singh J., Mia M., Pimenov D.Y., Chattopadhyaya S., Dwivedi S.P. (2020). Taguchi S/N and TOPSIS Based Optimization of Fused Deposition Modelling and Vapor Finishing Process for Manufacturing of ABS Plastic Parts. Materials.

[34] Prabhakaran S., Krishnaraj V., Sharma S., Senthilkumar M., Jegathishkumar R., Zitoune R. (2019). Experimental study on thermal and morphological analyses of green composite sandwich made of flax and agglomerated cork. J. Therm. Anal..

[35] Sharma S., Sudhakara P., Singh J., Ilyas R.A., Asyraf M.R.M., Razman M.R. (2021). Critical Review of Biodegradable and Bioactive Polymer Composites for Bone Tissue Engineering and Drug Delivery Applications. Polymers.

[36] Sharma S., Sudhakara P., Omran A.A.B., Singh J., Ilyas R.A. (2021). Recent Trends and Developments in Conducting Polymer Nanocomposites for Multifunctional Applications. Polymers.

[37] Jha K., Tyagi Y.K., Kumar R., Sharma S., Huzaifah M.R.M., Li C., Ilyas R.A., Dwivedi S.P., Saxena A., Pramanik A. (2021). Assessment of Dimensional Stability, Biodegradability, and Fracture Energy of Bio-Composites Reinforced with Novel Pine Cone. Polymers.

[38] Kadier A., Ilyas R.A., Huzaifah M.R.M., Harihastuti N., Sapuan S.M., Harussani M.M., Azlin M.N.M., Yuliasni R., Ibrahim R., Atikah M.S.N. (2021). Use of industrial wastes as sustainable nutrient sources for bacterial cellu-lose (BC) production: Mechanism, advances, and future perspectives. Polymers.

[39] Singh Y., Singh J., Sharma S., Lam T.-D., Nguyen D.-N. (2020). Fabrication and characterization of coir/carbon-fiber rein-forced epoxy based hybrid composite for helmet shells and sports-good applications: Influence of fiber surface modifications on the mechanical, thermal and morphological properties. J. Mater. Res. Technol..

[40] Suriani M.J., Ilyas R.A., Zuhri M.Y.M., Khalina A., Sultan M.T.H., Sapuan S.M., Ruzaidi C.M., Wan F.N., Zulkifli F., Harussani M.M. (2021). Critical review of natural fiber reinforced hybrid composites: Processing, properties, applications and cost. Polymers.

[41] 41. Kumar R., Ranjan N., Kumar V., Kumar R., Chohan J.S., Yadav A., Piyush, Sharma S., Prakash C., Singh S. (2021). Characterization of Friction Stir-Welded Polylactic Acid/Aluminum Composite Primed through Fused Filament Fabrication. J. Mater. Eng. Perform..

[42] Zhu Z., Wu Y., Liang Z. (2022). Mining-Induced Stress and Ground Pressure Behavior Characteristics in Mining a Thick Coal Seam with Hard Roofs. Front. Earth Sci..

[43] Azlin M.N.M., Ilyas R.A., Zuhri M.Y.M., Sapuan S.M., Harussani M.M., Sharma S., Nordin A.H., Nurazzi N.M., Afiqah A.N. (2022). 3D Printing and Shaping Polymers, Composites, and Nanocomposites: A Review. Polymers.

[44] Gu M., Mo H., Qiu J., Yuan J., Xia Q. (2022). Behavior of Floating Stone Columns Reinforced with Geogrid Encasement in Model Tests. Front. Mater..

[45] Kumar J., Singh D., Kalsi N.S., Sharma S., Pruncu C.I., Pimenov D.Y., Rao K.V., Kapłonek W. (2020). Comparative study on the mechanical, tribological, morphological and structural properties of vortex casting processed, Al-SiC-Cr hybrid metal matrix composites for high strength wear-resistant applications: Fabrication and characterizations. J. Mater. Res. Technol..

[46] Dwivedi S.P., Saxena A., Sharma S. (2021). Influence of Nano-CuO on Synthesis and Mechanical Behavior of Spent Alumina Catalyst and Grinding Sludge Reinforced Aluminum Based Composite. Int. J. Met..

[47] Dwivedi S.P., Saxena A., Sharma S., Srivastava A.K., Maurya N.K. (2021). Influence of SAC and Eggshell addition in the Physical, Me-chanical and Thermal Behaviour of Cr reinforced Aluminium Based Composite. Int. J. Cast Met. Res..

[48] Saxena A., Dwivedi S., Dixit A., Sharma S., Srivastava A., Maurya N. (2021). Computational and experimental investigation on mechanical behavior of zirconia toughened alumina and nickel powder reinforced EN31 based composite material. Mater. Werkst..

[49] Sharma S., Singh J., Gupta M.K., Mia M., Dwivedi S.P., Saxena A., Chattopadhyaya S., Singh R., Pimenov D.Y., Korkmaz M.E. (2021). Investigation on mechanical, tribological and microstruc-tural properties of Al-Mg-Si-T6/SiC/muscovite-hybrid metal-matrix composites for high strength applications. J. Mate-Rials Res. Technol..

[50] Dwivedi S.P., Agrawal R., Sharma S. (2021). Effect of Friction Stir Process Parameters on Mechanical Properties of Chrome Containing Leather Waste Reinforced Aluminium Based Composite. Int. J. Precis. Eng. Manuf. Technol..

[51] Kumar J., Singh D., Kalsi N.S., Sharma S., Mia M., Singh J., Rahman M.A., Khan A.M., Rao K.V. (2021). Investigation on the mechanical, tribological, morphological and machinability behavior of stir-casted Al/SiC/Mo reinforced MMCs. J. Mater. Res. Technol..

[52] Islam S., Dwivedi S.P., Dwivedi V.K., Sharma S., Kozak D. (2021). Development of Marble Dust/Waste PET Based Polymer Composite Ma-terial for Environmental Sustainability: Fabrication and Characterizations. J. Mater. Perform. Charact..

[53] Guo Y., Yang Y., Kong Z., He J., Wu H. (2022). Development of Similar Materials for Liquid-Solid Coupling and Its Application in Water Outburst and Mud Outburst Model Test of Deep Tunnel. Geofluids.

[54] Sharma S., Sudhakara P. (2019). Fabrication and optimization of hybrid AA-6082-T6 alloy/8%Al_2_O_3_(Alumina)/2%Grp metal matrix composites using novel Box-Behnken methodology processed by wire-sinking electric discharge machining. Mater. Res. Express.

[55] Dwivedi S.P., Saxena A., Sharma S., Singh G., Singh J., Mia M., Chattopadhyaya S., Pramanik A., Pimenov D.Y., Wojciechowski S. (2021). Effect of ball-milling process parameters on mechanical properties of Al/Al2O3/collagen powder composite using statistical approach. J. Mater. Res. Technol..

[56] Khare J.M., Dahiya S., Gangil B., Ranakoti L., Sharma S., Huzaifah M.R.M., Ilyas R.A., Dwivedi S.P., Chattopadhyaya S., Kilinc H.C. (2021). Comparative Analysis of Erosive Wear Behaviour of Epoxy, Polyester and Vinyl Esters Based Thermosetting Polymer Composites for Human Prosthetic Applications Using Taguchi Design. Polymers.

[57] Dwivedi S.P., Maurya M., Sharma S. (2021). Study of CCLW, Alumina and the Mixture of Alumina- and CCLW-Reinforced Aluminum-Based Composite Material with and Without Mechanical Alloying. J. Inst. Eng. Ser. D.

[58] Dwivedi S.P., Sahu R., Saxena A., Dwivedi V.K., Srinivas K., Sharma S. (2021). Recovery of Cr from chrome-containing leather waste and its utilization as reinforcement along with waste spent alumina catalyst and grinding sludge in AA 5052-based metal matrix composites. Proc. Inst. Mech. Eng. Part E J. Process. Mech. Eng..

[59] Dwivedi S.P., Maurya M., Saxena A., Sharma S. (2021). Synthesis and characterization of spent alumina catalyst and grinding sludge reinforced aluminium-based composite material. Proc. Inst. Mech. Eng. Part C J. Mech. Eng. Sci..

[60] Dwivedi S.P., Maurya M., Sharma S. (2022). Synthesis and characterisation of chromium, eggshell and grinding sludge-reinforced aluminium metal matrix composite: An experimental approach. Green Mater..

[61] Ilyas R.A., Aisyah H.A., Nordin A.H., Ngadi N., Zuhri M.Y.M., Asyraf M.R.M., Sapuan S.M., Zainudin E.S., Sharma S., Abral H. (2022). Natural-Fiber-Reinforced Chitosan, Chitosan Blends and Their Nanocomposites for Various Advanced Applications. Polymers.

[62] Asyraf M.R.M., Syamsir A., Zahari N.M., Supian A.B.M., Ishak M.R., Sapuan S.M., Sharma S., Rashedi A., Razman M.R., Zakaria S.Z.S. (2022). Product Development of Natural Fibre-Composites for Various Applications: Design for Sustainability. Polymers.

[63] Chandel P.S., Tyagi Y.K., Jha K., Kumar R., Sharma S., Singh J., Ilyas R.A. (2021). Study of mode II interlaminar fracture toughness of laminated composites of glass and jute fibres in epoxy for structural applications. Funct. Compos. Struct..

[64] Yeswanth I., Jha K., Bhowmik S., Kumar R., Sharma S., Rushdan A.I. (2022). Recent developments in RAM based MWCNT composite materials: A short review. Funct. Compos. Struct..

[65] Virk G.S., Singh B., Singh Y., Sharma S., Ilyas R.A., Patyal V. (2022). Abrasive water jet machining of coir fiber reinforced epoxy composites: A review. Funct. Compos. Struct..

[66] Juneja S., Chohan J.S., Kumar R., Sharma S., Ilyas R.A., Asyraf M.R.M., Razman M.R. (2022). Impact of Process Variables of Acetone Vapor Jet Drilling on Surface Roughness and Circularity of 3D-Printed ABS Parts: Fabrication and Studies on Thermal, Morphological, and Chemical Characterizations. Polymers.

[67] Singh S., Khairandish M.I., Razahi M.M., Kumar R., Chohan J.S., Tiwary A., Sharma S., Li C., Ilyas R.A., Asyraf M.R.M. (2022). Preference Index of Sustainable Natural Fibers in Stone Matrix Asphalt Mixture Using Waste Marble. Materials.

[68] Tiwary A.K., Singh S., Chohan J.S., Kumar R., Sharma S., Chattopadhyaya S., Abed F., Stepinac M. (2022). Behavior of RC Beam–Column Joints Strengthened with Modified Reinforcement Techniques. Sustainability.

[69] Tiwary A.K., Bhatia S., Singh S., Chohan J.S., Kumar R., Sharma S., Chattopadhyaya S., Rajkumar S. (2022). Performance Comparison and Critical Finite Element Based Experimental Analysis of Various Forms of Reinforcement Retaining Structural System. Math. Probl. Eng..

[70] Ranakoti L., Gangil B., Mishra S.K., Singh T., Sharma S., Ilyas R., El-Khatib S. (2022). Critical Review on Polylactic Acid: Properties, Structure, Processing, Biocomposites, and Nanocomposites. Materials.

[71] Norfarhana A., Ilyas R., Ngadi N., Sharma S., Sayed M.M., El-Shafay A., Nordin A. (2022). Natural Fiber-Reinforced Thermoplastic ENR/PVC Composites as Potential Membrane Technology in Industrial Wastewater Treatment: A Review. Polymers.

[72] Tiwary A.K., Singh S., Kumar R., Chohan J.S., Sharma S., Singh J., Li C., Ilyas R.A., Asyraf M.R.M., Malik M.A. (2022). Effects of Elevated Temperature on the Residual Behavior of Concrete Containing Marble Dust and Foundry Sand. Materials.

[73] Robert J.B., Prabhavathy R.A., Joanna P.S., Singh S.C.E., Murugan S., Rajkumar S., Sharma S. (2021). Flexural Behaviour of RC Beams with a Circular Opening at the Flexural Zone and Shear Zone Strengthened Using Steel Plates. Adv. Civ. Eng..

